# Influence of a complex mycotoxin detoxifier on growth performance, liver health, inflammation, and redox status of broilers fed a diet contaminated with multiple mycotoxins

**DOI:** 10.1371/journal.pone.0354753

**Published:** 2026-07-30

**Authors:** Nampetch Puengpong, Thaweesak Songserm, Konkawat Rassmidatta, Joan Torrent, Alireza Khadem, Arnau Vidal, Christos Gougoulias, Nithikarn Chanaksorn, Kazeem D. Adeyemi, Yuwares Ruangpanit

**Affiliations:** 1 Department of Animal Science, Faculty of Agriculture at Kamphaeng Saen, Kasetsart University, Kamphaeng Saen Campus, Nakhon Pathom, Thailand; 2 Department of Pathology, Faculty of Veterinary Medicine, Kasetsart University, Bangkok, Thailand; 3 Innovad Group, Berchem, Belgium; 4 Laboratory of Nutrition, Faculty of Veterinary Medicine, Ghent University, Merelbeke, Belgium; 5 Innovad (Thailand) Co., Ltd, Bangplee, Samutprakarn, Thailand; 6 Department of Animal Production, Faculty of Agriculture, University of Ilorin, Ilorin, Kwara State, Nigeria; Sindh Agriculture University, PAKISTAN

## Abstract

This study investigated the effects of a complex mycotoxin detoxifier (CMD) on growth performance, liver health, inflammation, and redox status of broilers fed a diet contaminated with multiple mycotoxins. A total of 432 one-day-old male Ross 308 broiler chicks were randomly allocated to one of three treatments, with eight replicates per dietary group for 37 d. The treatment included: a basal diet without mycotoxin, positive control, PC; a basal diet contaminated with 80 ppb Aflatoxin + 900 ppb fumonisins + 30 ppb deoxynivalenol + 80 ppb Zearalenone, negative control, NC or NC supplemented with CMD (sodium bentonite, sepiolite, yeast cell wall, and β-glucans) at 1 and 0.5 kg/ton in the starter and finisher diet respectively. Compared with the PC and CMD groups, NC birds showed higher feed intake and poorer feed efficiency during days 1–10 (p < 0.05), although these differences were not observed thereafter. Newcastle disease antibody titer was reduced in NC birds but restored by CMD supplementation (p < 0.05). Birds receiving CMD also exhibited higher IL-10 concentrations than both PC and NC birds (p < 0.05). Furthermore, CMD supplementation reduced macroscopic fatty liver scores and liver inflammation (histopathological lesions) compared with the NC group (p < 0.05). In conclusion, dietary exposure to multiple mycotoxins caused only transient impairment of growth performance but adversely affected immune response and liver health. Supplementation with CMD at 1.0 and 0.5 kg/ton during the starter and finisher phases, respectively, alleviated several of these adverse effects in broiler chickens.

## 1. Introduction

The poultry industry remains a cornerstone of global food security by providing a high-quality source of animal protein to a growing human population [1]. Global poultry meat production has continued to increase steadily over the past decades and recently exceeded 140 million metric tons, reflecting the growing global demand for poultry products [2,3]. However, the efficiency and profitability of poultry production are increasingly compromised by feed contamination [4,5]. Feed contamination refers to the presence of harmful biological, chemical, or environmental substances in feed ingredients that compromise animal health, productivity, and the safety of animal-derived products [6,7]. Among these contaminants, mycotoxins, which are secondary metabolites produced by fungal species such as *Aspergillus*, *Fusarium*, and *Penicillium* [8,9], are considered one of the most important due to their widespread occurrence and persistence in feedstuffs.

Mycotoxin contamination is widespread in both tropical and temperate regions, often exacerbated by poor storage conditions, climate variability, environmental stressors, and poor agricultural practices [10,11]. In poultry, chronic exposure to mycotoxins impairs feed efficiency, suppresses growth performance, disrupts intestinal integrity, and compromises immune competence [8,9,12]. The immunosuppressive effects of mycotoxins are mediated through several mechanisms, including inhibition of lymphocyte proliferation, dysregulation of cytokine production, suppression of antibody synthesis, oxidative damage to immune cells, and disruption of gut barrier function [13–15]. These alterations reduce vaccine responsiveness and increase susceptibility to infectious diseases in broiler chickens [15].

Beyond immunotoxicity, mycotoxins profoundly affect systemic metabolism, particularly hepatic lipid metabolism and redox homeostasis. Exposure to aflatoxins, fumonisins, deoxynivalenol (DON), and zearalenone (ZEN) has been associated with disturbances in lipid synthesis, fatty acid oxidation, lipoprotein transport, and mitochondrial energy metabolism, ultimately promoting hepatic steatosis and metabolic dysfunction [16,17]. These toxins disrupt lipid homeostasis by inducing oxidative stress, activating inflammatory signaling pathways, impairing mitochondrial function, and dysregulating the expression of genes involved in lipid synthesis and β-oxidation, thereby contributing to systemic metabolic disorders in chickens [18,19]. Furthermore, because the liver is the primary organ responsible for detoxification, biotransformation, and nutrient metabolism, it is particularly susceptible to mycotoxin-induced injury. Mycotoxins can impair hepatic drug-metabolizing enzymes and membrane transport systems, reducing xenobiotic clearance while promoting hepatocellular damage through oxidative stress, apoptosis, and inflammation [16,19]. Collectively, these alterations disrupt hepatic metabolic and transport functions, exacerbating liver injury and systemic metabolic imbalance [16,17,20]. At the molecular level, mycotoxin-induced liver injury is strongly associated with excessive reactive oxygen species (ROS) production, lipid peroxidation, mitochondrial dysfunction, and activation of pro-inflammatory pathways such as nuclear factor-kappa B and mitogen-activated protein kinase signaling [18,19,21]. These pathways contribute to hepatocyte degeneration, inflammatory infiltration, and impaired nutrient utilization, which collectively reduce broiler productivity and health status [18,19,21].

To mitigate these adverse effects, complex mycotoxin detoxifiers (CMDs) have gained considerable attention [22–25]. These products commonly contain clay minerals, yeast cell wall components, and bioactive plant compounds [22–26]. Components such as sodium bentonite [26–28], sepiolite [26], yeast cell walls [29–32], and β-glucans [33] have been reported to reduce mycotoxin bioavailability, improve growth performance, and mitigate oxidative stress and inflammatory responses in broiler chickens. Their primary mode of action involves adsorbing mycotoxins in the gastrointestinal tract, thereby reducing their absorption and systemic toxicity [27,28]. In addition, many bioactive plant compounds possess antioxidant, anti-inflammatory, and gut-protective properties [34–37], making them particularly beneficial under conditions of multiple stressors. This multifaceted mode of action is especially important because multiple mycotoxins frequently co-occur in feed and can exert additive or synergistic toxic effects that are more severe than those induced by individual toxins alone [23,29]. For example, combined exposure to zearalenone, aflatoxin B1, deoxynivalenol, ochratoxin A, and T-2 toxin has been shown to reduce growth performance and impair intestinal health in broilers [32]. Similarly, concurrent exposure to aflatoxins, zearalenone, T-2 toxin, fumonisins, and deoxynivalenol compromised gut integrity and intestinal function [22]. Furthermore, co-exposure to deoxynivalenol and its acetylated derivatives, 3-acetyl-deoxynivalenol and 15-acetyl-deoxynivalenol, reduced growth performance and caused intestinal damage in broiler chickens [38]. Therefore, evaluating detoxification strategies under multiple-mycotoxin exposure conditions is more representative of commercial poultry production systems.

Despite increasing evidence supporting the efficacy of mycotoxin detoxifiers, little is known about the ability of complex mycotoxin detoxifiers to alleviate liver injury, inflammatory responses, and oxidative imbalance under practical multiple-mycotoxin exposure conditions in broiler chickens. We hypothesized that dietary supplementation with a complex mycotoxin detoxifier containing sodium bentonite, sepiolite, yeast cell wall components, and β-glucans would alleviate the adverse effects of combined aflatoxin, fumonisin, deoxynivalenol, and zearalenone contamination by significantly improving growth performance, preserving liver histopathology, reducing inflammatory responses, and enhancing antioxidant status in broiler chickens compared with birds fed contaminated diets without the detoxifier. Therefore, the objective of this study was to evaluate the effects of a complex mycotoxin detoxifier on growth performance, liver health, immune response, inflammation, and oxidative status in broilers fed diets contaminated with multiple mycotoxins.

## 2. Materials and methods

### 2.1. Biosafety measures during experimental procedures

In this experiment, all personnel were required to wear complete personal protective equipment (PPE) throughout procedures involving feed or materials potentially contaminated with aflatoxins, in accordance with standard laboratory practices that classify mycotoxins as biohazardous substances. The PPE included nitrile gloves to prevent direct dermal contact; safety goggles or face shields to protect against airborne particles or splashes; and disposable masks or N95 particulate respirators to reduce inhalation of contaminated dust generated during the handling or sorting of feed materials. In addition, personnel wore laboratory coats, gowns, or disposable protective suits over personal clothing, as well as impermeable footwear or shoe covers, to minimize cross-contamination between work areas. All PPE was removed prior to leaving the work area, followed by thorough handwashing. All procedures were conducted in adequately ventilated environments to further reduce exposure risks.

Waste generated during the experiment including leftover feed, residual materials, and excreta suspected of aflatoxin contamination, was collected in designated, tightly sealed containers clearly labeled as hazardous waste and disposed of through the institution’s certified hazardous waste management system. At the conclusion of the experiment, broiler carcasses were treated as biological waste and disposed of through controlled burial within designated areas. All procedures were conducted in accordance with internationally recognized biosafety and occupational health guidelines to minimize the risks associated with aflatoxin exposure to humans, animals, and the environment [39–42].

### 2.2. Aflatoxin production

The fungal cultures were obtained from the Department of Plant Pathology at Kasetsart University, Kamphaeng Saen Campus, Nakhon Pathom, Thailand. Aflatoxin was produced by cultivating *Aspergillus flavus* A17 in darkness at 28–30°C for 14 days. After 14 days, A. flavus A17 spores were harvested from PDA agar and transferred into a test tube containing 10 mL of a 0.1% Tween 20 solution. Separately, 500 g of corn (mesh size: 2.00 mm) was combined with 40 mL of water and left overnight at 4°C. The mixture was then autoclaved. The prepared spore suspension was added to the sterilized corn mixture, followed by incubation at 28–30°C for 14 days. After incubation, the mixture was autoclaved and further dried at 104°C for 8 h.

### 2.3. Ethical approval

All animal care and experimental procedures were approved by the Institutional Animal Care and Use Committee of Kasetsart University, Thailand (Approval No. ACKU66-AGK-029; 8 September 2023), and conducted in accordance with the university’s Regulations on Animal Experiments and the ARRIVE Guidelines 2.0. Birds were monitored daily and predefined humane endpoints were applied. Broilers exhibiting severe distress, inability to access feed or water, persistent recumbency, marked respiratory distress, severe weight loss, neurological symptoms, or severe injury or illness were euthanized immediately upon detection using CO_2_ inhalation. A small number of birds died during the experiment and were recorded as mortality in the performance data. All birds were euthanized by CO_2_ inhalation at the end of the 37-day trial.

### 2.4. Birds, diets, and management

A completely randomized design was employed in this study. A total of 432 one-day-old male Ross 308 broiler chicks were randomly allocated to three dietary treatments, each consisting of eight replicate pens with 18 birds per pen (144 birds per treatment). The pen served as the experimental unit. The experiment lasted 37 days.

The sample size was selected based on previous broiler nutrition and mycotoxin challenge studies employing similar experimental designs and numbers of replicates to provide sufficient statistical power for detecting biologically meaningful differences among treatments while accounting for expected within-treatment variability. The treatments were as follows:

- Positive control (PC): Basal diet without mycotoxin contamination.
- Negative control (NC): Basal diet contaminated with 80 ppb aflatoxin, 900 ppb fumonisins, 30 ppb deoxynivalenol, and 80 ppb zearalenone.
- CMD: NC diet supplemented with a complex mycotoxin detoxifier (CMD; Magnet®, Innovad NV/SA, Postbaan 69, 2910 Essen, Belgium), comprising sodium bentonites, sepiolite, yeast cell wall components, and β-glucans.

The CMD was added at 1 kg/ton of feed during the starter phase and 0.5 kg/ton during the grower and finisher phases. The concentrations of mycotoxins in all experimental diets were maintained below current regulatory or guidance values. Specifically, the aflatoxin concentration was below the maximum limit established by the Department of Livestock Development, Thailand [43], whereas fumonisin, deoxynivalenol, and zearalenone concentrations were below the guidance values established by the European Commission [44]. These concentrations were selected to simulate the low-level multi-mycotoxin contamination frequently encountered under commercial field conditions. Although individual toxin levels were below regulatory thresholds, chronic exposure to multiple co-occurring mycotoxins may still induce additive or synergistic biological effects. Therefore, the study was designed to evaluate whether subclinical contamination could impair broiler performance and whether supplementation with a complex mycotoxin detoxifier could mitigate such effects.

**Fig 1:**
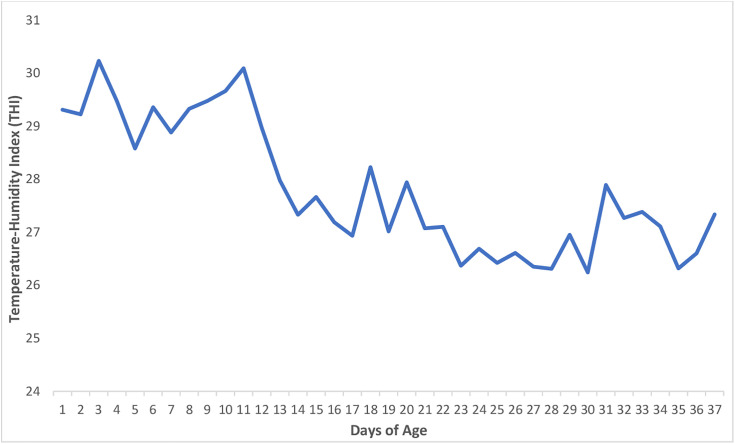
Daily Temperature-Humidity Index recorded throughout the experimental period.

All birds were housed in an environmentally controlled facility and raised on floor pens bedded with rice hulls. The daily Temperature-Humidity Index (THI) recorded throughout the experimental period is presented in Fig 1. Diets were formulated according to Ross 308 nutrient recommendations [45] (Table 1) and provided in mash form. Feed and water were supplied *ad libitum* throughout the 37-day study, which followed a three-phase feeding program (starter, grower, and finisher). Birds were vaccinated against Newcastle disease (B1 strain) on day 7, infectious bursal disease (IB) on day 14, and Newcastle disease (LaSota strain) and IB on day 21. The chemical composition of the feed was analyzed according to AOAC [46] methods and is presented in Table 2.

**Table 1:** Ingredients and chemical composition of diets.

Ingredient (%)		Starter (d 1–10)		Grower (d 11–24)		Finisher (d 25–37)	
		PC	NC	PC	NC	PC	NC
Normal Corn		57.1	26.8	61.37	30.85	63.64	33.34
Mycotoxin-contaminated Corn		–	30	–	30	–	30
Dehulled Soybean meal (48% Crude protein)		35.79	35.66	29.42	29.5	24.93	24.8
Full fat soybean		3.00	3.00	5.00	5.00	7.00	7.00
Soybean oil		0.02	0.47	0.51	0.99	1.33	1.77
Monocalcium phosphate		1.29	–	1.07	1.07	0.84	0.84
Limestone		1.16	1.14	1.06	1.04	0.95	0.93
Salt		0.04	0.04	0.07	0.07	0.11	0.11
Premix^1^		0.20	0.20	0.20	0.20	0.20	0.20
DL-Methionine		0.32	0.32	0.28	0.28	0.24	0.24
L-Lysine HCl		0.22	0.12	0.22	0.21	0.15	0.16
L-Threonine		0.12	0.02	0.09	0.09	0.05	0.05
L-Valine		0.02	1.30	0.02	0.02	–	–
L-Isoleucine		–	0.22	–	–	–	–
Sodium bicarbonate		0.36	0.36	0.32	0.31	0.25	0.25
Choline Chloride 60%		0.09	0.09	0.09	0.09	0.09	0.09
Antimold		0.20	0.20	0.20	0.20	0.20	0.20
Coccidiostat (Salinomycin 12%)		0.05	0.05	0.05	0.05	–	–
Phytase (Quantum Blue 5G)		0.01	0.01	0.01	0.01	0.01	0.01
Calculated Chemical Composition							
Dry matter	%	87.67	90.33	87.67	90.33	87.7	90.37
Crude protein	%	23.00	23.00	21.50	21.50	19.50	19.50
Crude fat	%	3.26	4.15	4.20	5.12	5.41	6.30
Crude fiber	%	4.08	3.56	3.94	3.43	3.85	3.34
Ash	%	5.67	5.58	5.16	5.08	4.73	4.64
ME for poultry	Kcal/Kg	3,000	3,000	3,100	3,100	3,200	3,200
Dig.Lys (poultry)	%	1.28	1.28	1.15	1.15	1.02	1.02
Dig.Met (poultry)	%	0.63	0.63	0.57	0.57	0.51	0.51
Calcium	%	0.96	0.96	0.87	0.87	0.78	0.78
Phosphorus-total	%	0.67	0.67	0.60	0.60	0.54	0.53
Phosphorus-avail	%	0.48	0.48	0.44	0.44	0.39	0.39
Sodium	%	0.16	0.16	0.16	0.16	0.16	0.16
Chloride	%	0.23	0.23	0.23	0.23	0.23	0.23

PC: basal diet without mycotoxin contamination; NC: basal diet contaminated with 80 ppb Aflatoxin + 900 ppb fumonisins + 30 ppb deoxynivalenol + 80 ppb Zearalenone; CMD: NC + complex mycotoxin detoxifier. ^1^Premix contains minerals and vitamins per kg premix, including Vitamin A 6,000,000 IU, Vitamin D3 1,200,000 IU, Vitamin E 30.00 g, Vitamin K 1.50 g, Vitamin B1 1.50 g, Vitamin B2 4.00 g, Vitamin B6 2.00 g, Vitamin B12 0.01 g, Niacin 25.00 g, Pantothenic acid 7.50 g, Biotin 0.20 g, Folic acid 1.00 g, Copper 7.50 g, Iron 20.00 g, Manganese 50.00 g, Zinc 50.00 g, Iodine 0.50 g, Selenium 0.15 g, Antioxidant 2.50 g, Filler (rice hull ground only) 554.29 g.

**Table 2:** Analyzed nutrient composition of dietary treatments.

	Dietary treatment^1^		
Nutrient	PC	NC	CMD
Starter			
Dry matter (%)	88.24	88.28	88.67
Protein (%)	22.56	22.51	22.59
Fat (%)	3.52	3.51	3.53
Fiber (%)	2.68	2.63	2.65
Ash (%)	5.33	5.36	5.35
Calcium (%)	0.83	0.83	0.84
Total phosphorus (%)	0.66	0.65	0.67
Gross energy (kcal/kg)	3,945	3,946	3,956
Grower			
Dry matter (%)	88.44	88.81	88.51
Protein (%)	20.71	20.73	20.72
Fat (%)	4.49	4.46	4.47
Fiber (%)	2.46	2.44	2.44
Ash (%)	4.80	4.81	4.82
Calcium (%)	0.75	0.77	0.76
Total phosphorus (%)	0.59	0.60	0.59
Gross energy (kcal/kg)	4,049	4,049	4,047
Finisher			
Dry matter (%)	88.39	88.31	88.28
Protein (%)	19.19	19.16	19.11
Fat (%)	5.96	5.93	5.94
Fiber (%)	2.43	2.48	2.45
Ash (%)	4.26	4.29	4.25
Calcium (%)	0.67	0.65	0.66
Total phosphorus (%)	0.54	0.52	0.53
Gross energy (kcal/kg)	4,075	4,072	4,072

^1^PC: basal diet without mycotoxin contamination; NC: basal diet contaminated with 80 ppb Aflatoxin + 900 ppb fumonisins + 30 ppb deoxynivalenol + 80 ppb Zearalenone; CMD: NC + complex mycotoxin detoxifier.

### 2.5. Feed mycotoxin analysis

#### 2.5.1. Sample extraction.

Feed samples (5 g) were extracted with 20 mL of acetonitrile/water/acetic acid (79:20:1, v/v/v) using an overhead shaker for 1 h, followed by centrifugation at 3,300 × g for 15 min. Supernatants were purified on Strata C18-E SPE columns (Phenomenex, Utrecht, The Netherlands) preconditioned with extraction solvent. Eluates were pooled, adjusted to 25 mL, and defatted with n-hexane. Defatted extracts were divided for two clean-up procedures: (i) filtration through Whatman glass microfilters and (ii) passage through MultiSep 226 AflaZon+ columns (Romer Labs, The Netherlands). Purified extracts were combined, evaporated under nitrogen, reconstituted in 150 µL of mobile phase, centrifuged through 0.22 µm PVDF filters, and transferred to HPLC vials for analysis.

#### 2.5.2. UHPLC–MS/MS analysis.

Chromatographic separation and detection were performed using a Waters Acquity UPLC system coupled with a Quattro Premier XE triple quadrupole mass spectrometer (Waters, Zellik, Belgium). Separation was achieved on a Symmetry C18 column (5 µm, 150 × 2.1 mm) with a guard column of the same chemistry. Injections of 10 µL were made at 25 °C using a gradient elution of mobile phase A (water/methanol/acetic acid, 94:5:1, v/v/v with 5 mM ammonium acetate) and mobile phase B (water/methanol/acetic acid, 2:97:1, v/v/v with 5 mM ammonium acetate) at 0.3 mL/min for 30 min.

The MS was operated in positive electrospray ionization (ESI+) mode, with source and desolvation temperatures of 120 °C and 400 °C, respectively, capillary voltage of 20 kV, and nitrogen as spray gas (cone gas 50 L/h; desolvation gas 800 L/h). Data were acquired in multiple reaction monitoring mode. Data acquisition and processing were conducted with MassLynx™ and QuanLynx® software (v4.1, Waters, UK). The mycotoxin contents of the finished feed are presented in Table 3.

**Table 3:** Mycotoxin contents of finished feed.

Mycotoxin (ppb)	Dietary treatment^1^		
	PC	NC	CMD
15 DOA			
Aflatoxin B1	–	94.10	87.70
Aflatoxin B2	–	5.60	5.40
Deoxynivalenol	–	80.00	33.00
Fumonisin B1	–	999.00	998.00
Zearalenone	–	83.70	124.00
37 DOA			
Aflatoxin B1	–	86.80	109.00
Aflatoxin B2	–	5.00	5.90
Deoxynivalenol	–	48.00	45.00
Fumonisin B1	63.00	1,080.00	1,640.00
Zearalenone	–	173.00	150.00

^1^PC: basal diet without mycotoxin contamination; NC: basal diet contaminated with 80 ppb Aflatoxin + 900 ppb fumonisins + 30 ppb deoxynivalenol + 80 ppb Zearalenone; CMD: NC + complex mycotoxin detoxifier.

### 2.6. Growth performance

The body weight of all birds in each pen was individually recorded on days 1, 10, 24, and 37 to calculate periodical body weight gain (BWG). Feed intake (FI) was calculated from the difference between feed offered and feed refused per replicate. The feed conversion ratio (FCR) was subsequently derived from these data. Mortality was recorded daily throughout the experimental period to determine overall survival rate.

### 2.7. Inflammation and oxidative stress

On days 15 and 37, two birds per pen were randomly selected for blood collection via wing vein puncture. Serum samples were analyzed to quantify malondialdehyde [47], glutathione (GSH; MyBiosource, Chicken Glutathione ELISA Kit, Cat. No. MBS263343, San Diego, USA), interleukin 6 (IL-6; MyBiosource, Chicken Interleukin 6 ELISA Kit, Cat. No. MBS288783, San Diego, USA), and interleukin 10 (IL-10; MyBiosource, Chicken Interleukin 10 ELISA Kit, Cat. No. MBS701683, San Diego, USA).

### 2.8. Liver health and enzymes

The severity of fatty liver was assessed on day 37 by euthanizing two birds per pen, excising the liver, and evaluating liver coloration, pathological lesions, histological features, and macroscopic alterations (including inflammation and hemorrhage), following the procedures described by Choi et al. [48]. Histological slides were examined under 400 × magnification using an optical microscope, according to Malisorn et al. [49]. Serum aspartate aminotransferase (AST), alanine aminotransferase (ALT), and gamma-glutamyl transferase (GGT) activities were determined using the QuantILab® ALT/GPT Liquid method [50].

### 2.9. Statistical analysis

Data were evaluated for normality using the Shapiro–Wilk test, and homogeneity of variances was assessed using Levene’s test. All data, except liver fatty scores, were analyzed using the General Linear Model procedure of SAS (version 9.4; SAS Institute Inc., Cary, NC, USA) according to the following model for a completely randomized design:


$$\begin{array}{c} {\text{Y}}_{\text{ij}}=\mu +{\text{T}}_{\text{i}}+{\text{e}}_{\text{ij}} \end{array}$$

where:

Y_ij_ is the observed response variable, μ is the overall mean, T_i_ is the fixed effect of the (i)th dietary treatment, and e_ij_ is the random residual error.

When the F test is significant, treatment means were separated using Tukey’s honestly significant difference test, with statistical significance declared at p < 0.05. Liver fatty scores were analyzed by Kruskal–Wallis test. A pen of 18 birds was the experimental unit.

## 3. Results and discussion

### 3.1. Growth performance

From 1 to 10 days, BWG was not significantly (P = 0.899) affected by mycotoxin contamination (Table 4). However, birds in the NC group (exposed to mycotoxins without a detoxifier) consumed more feed (P = 0.020) and had a poorer FCR (P = 0.039) than those in the PC group during days 1–10. (Table 4). Aflatoxins and DON are known to impair intestinal barrier integrity by disrupting tight junction proteins, increasing epithelial permeability, and reducing nutrient absorption efficiency, whereas fumonisins interfere with sphingolipid metabolism and intestinal cell membrane integrity [16,51]. These alterations may reduce digestive efficiency and contribute to compensatory increases in feed intake. Consistent with these findings, broilers raised on diets contaminated with aflatoxin B1 [8,9], fumonisins [9], ochratoxin A and T-2 [52], and deoxynivalenol [31,53] exhibited reduced feed efficiency. Conversely, feeding diets contaminated with multiple mycotoxins including T-2 toxin, aflatoxin B1, ochratoxin A, deoxynivalenol, and zearalenone reduced BWG in broilers [32].

**Table 4:** Growth performance in broilers fed mycotoxin-contaminated diet and supplemented with a complex mycotoxin detoxifier.

	Dietary treatment^1^				
Parameter	PC	NC	CMD	SEM	*P*-value
Initial weight (g)	39.44	39.48	39.13	0.13	0.484
0-10 DOA					
Body weight gain (g)	222.25	221.29	221.13	1.02	0.899
Feed intake (g)	261.67^b^	277.46^a^	270.19^ab^	2.41	0.020
Feed conversion ratio	1.18^a^	1.25^b^	1.22^ab^	0.01	0.039
Mortality (%)	0.00	0.00	0.00		
11-24 DOA					
Body weight gain (g)	810.09	808.53	821.86	5.06	0.522
Feed intake (g)	1134	1134	1143	7.59	0.864
Feed conversion ratio	1.40	1.40	1.39	0.01	0.823
Mortality (%)	0.00	0.00	0.00		
25-37 DOA					
Body weight gain (g)	1261	1213	1250	16.88	0.497
Feed intake (g)	2035	1969	2025	0.02	0.976
Feed conversion ratio	1.61	1.62	1.62	0.02	0.977
Mortality (%)	0.00	0.78	0.74	0.56	0.800
1-37 DOA					
Body weight gain (g)	2293	2243	2293	20.53	0.532
Feed intake (g)	3431	3380	3438	21.32	0.502
Feed conversion ratio	1.49	1.51	1.50	0.01	0.871
Mortality (%)	0.00	0.96	0.89	0.68	0.800

a, b Means within the same column with different superscripts differ significantly (P < 0.05).

^1^PC: basal diet without mycotoxin contamination; NC: basal diet contaminated with 80 ppb Aflatoxin + 900 ppb fumonisins + 30 ppb deoxynivalenol + 80 ppb Zearalenone; CMD: NC + complex mycotoxin detoxifier

During this initial phase, FI and FCR of birds in the CMD treatment, which received the mycotoxin detoxifier, did not differ from those of the other two groups. Compared with NC birds, CMD supplementation numerically reduced FI from 277.46 to 270.19 g and improved FCR from 1.25 to 1.22 during days 1–10, indicating partial mitigation of the adverse effects of mycotoxin exposure. The effectiveness of mycotoxin detoxifiers depends on several factors, including toxin type and mode of action. Supporting this, Srinual et al. [32] observed that supplementing *Sporidiobolus pararoseus* in multi-mycotoxin-contaminated diets partially improved FCR in broilers. Similarly, yeast cell wall supplementation has been shown to mitigate the negative effects of multi-mycotoxin contamination on FCR in broilers [38]. Beyond 10 days of age, no significant differences in FI, BWG, FCR, or mortality were observed among treatments during the 11–25, 25–37, and 1–37-day periods. This suggests that broilers either adapted to mycotoxin exposure over time or exhibited compensatory growth.

### 3.2. Oxidative stress and inflammation

On day 15, IL-10 concentration in CMD birds increased (P = 0.004) by approximately 18.3% and 23.2% compared with the PC and NC birds, respectively (Table 5). Similarly, on day 37, IL-10 increased (P = 0.001) by approximately 21.8% and 24.5% relative to the PC and NC groups. IL-10 is a potent anti-inflammatory cytokine that regulates immune homeostasis by suppressing excessive pro-inflammatory responses and limiting tissue damage. The elevated IL-10 concentrations observed in CMD birds suggest that supplementation with the complex mycotoxin detoxifier enhanced anti-inflammatory immune regulation and mitigated the immunomodulatory effects of dietary mycotoxins. This effect may be attributed to the yeast-derived β-glucans and yeast cell wall components in the detoxifier, which interact with pattern recognition receptors on immune cells, thereby promoting macrophage activation, improving innate immune function, and stimulating the production of regulatory cytokines such as IL-10 [17,23]. In addition, the clay minerals (sodium bentonite and sepiolite) can adsorb mycotoxins within the gastrointestinal tract, reducing their bioavailability and subsequent inflammatory challenge [26–28]. These mechanisms collectively may contribute to the enhanced anti-inflammatory status observed in CMD-supplemented birds. Furthermore, anti-inflammatory interventions have been shown to attenuate inflammatory damage by suppressing endoplasmic reticulum stress and inflammatory signaling pathways, including NF-κB, providing additional mechanistic support for the immunomodulatory effects observed in the present study [54].

**Table 5:** Immune response and oxidative stress in broilers fed mycotoxin-contaminated diet and supplemented with a complex mycotoxin detoxifier.

	Dietary treatment^1^				
Parameter	PC	NC	CMD	SEM	*P*-value
Day 15					
Interleukin					
IL-6 (pg/ml)	447.91	563.30	474.40	43.24	0.643
IL-10(pg/ml)	2.78^b^	2.67^b^	3.29^a^	0.08	0.004
Oxidative stress					
Glutathione (pg/ml)	35.94	30.92	31.67	1.69	0.392
Malondialdehyde (µmol/ml)	10.85	10.91	10.88	0.15	0.991
Day 37					
Interleukin					
IL-6 (pg/ml)	178.33	218.41	181.64	12.61	0.525
IL-10 (pg/ml)	3.71^b^	3.63^b^	4.52^a^	0.12	0.001
Oxidative stress					
Glutathione (pg/ml)	36.14	32.02	34.92	2.57	0.534
Malondialdehyde (µmol/ml)	11.24	11.59	10.83	0.23	0.412

a, b Means within the same column with different superscripts differ significantly (P < 0.05).

^1^PC: basal diet without mycotoxin contamination; NC: basal diet contaminated with 80 ppb Aflatoxin + 900 ppb fumonisins + 30 ppb deoxynivalenol + 80 ppb Zearalenone; CMD: NC + complex mycotoxin detoxifier.

However, neither mycotoxin contamination nor CMD supplementation affected IL-6, GSH, or MDA concentrations on days 15 and 37. IL-6 is a key pro-inflammatory cytokine that is typically upregulated during acute inflammatory responses. The absence of changes in IL-6 may reflect the relatively low concentrations of mycotoxins used in the present study, which were below regulatory guidance values and may not have been sufficient to elicit a robust systemic inflammatory response. Alternatively, the concurrent increase in the anti-inflammatory cytokine IL-10 in CMD-supplemented birds may have contributed to maintaining immune homeostasis by limiting excessive pro-inflammatory cytokine production. Previous studies have also reported inconsistent effects of aflatoxins on IL-6 expression, with reductions or no changes observed depending on mycotoxin dose, duration of exposure, and animal species [55,56], highlighting the complex nature of mycotoxin-induced immune modulation.

Likewise, GSH and MDA, established indicators of antioxidant status and lipid peroxidation, remained unchanged despite dietary mycotoxin exposure. Although mycotoxins are known to induce oxidative stress through excessive reactive oxygen species generation, the low-level chronic contamination used in this study may have been insufficient to overwhelm endogenous antioxidant defenses and cause measurable oxidative damage. Furthermore, compensatory antioxidant mechanisms may have preserved redox balance, thereby preventing depletion of GSH and accumulation of MDA. These findings suggest that the biological effects of low-dose multi-mycotoxin exposure were manifested primarily through subtle immunomodulatory changes rather than overt oxidative stress, emphasizing that immune biomarkers may be more sensitive indicators of subclinical mycotoxin challenge under field-relevant conditions.

### 3.3. Newcastle disease titer

The NC treatment resulted in significantly lower (P = 0.030) ND titers than the PC and CMD treatments (Table 6), indicating that mycotoxin contamination impaired the immune response to ND vaccination. Mycotoxins, particularly aflatoxins and fumonisins, are well-documented immunosuppressants that inhibit lymphocyte proliferation, impair B-cell function and antibody production, disrupt antigen presentation, and alter cytokine signaling, ultimately reducing the efficacy of vaccine-induced humoral immunity [56–58]. Reduced ND titers in NC birds therefore suggest that chronic exposure to multiple mycotoxins compromised adaptive immune function, rendering birds more susceptible to Newcastle disease and other infectious challenges. Similar findings have been reported in broilers fed multi-mycotoxin-contaminated diets [59–61].

**Table 6:** ND titer in broilers fed mycotoxin-contaminated diet and supplemented with a complex mycotoxin detoxifier.

	Dietary treatment^1^				
Parameter	PC	NC	CMD	SEM	*P* value
ND titer	1.87^a^	1.56^b^	1.96^a^	0.06	0.030

a, b Means within the same column with different superscripts differ significantly (P < 0.05).

^1^PC: basal diet without mycotoxin contamination; NC: basal diet contaminated with 80 ppb Aflatoxin + 900 ppb fumonisins + 30 ppb deoxynivalenol + 80 ppb Zearalenone; CMD: NC + complex mycotoxin detoxifier.

In contrast, ND titers in CMD birds were comparable to those in PC birds, suggesting that supplementation with the complex mycotoxin detoxifier mitigated the immunosuppressive effects of mycotoxins. The inclusion of sodium bentonites, sepiolite, yeast cell wall components, and β-glucans likely reduced mycotoxin bioavailability and systemic absorption. Yeast-derived β-glucans, in particular, are known to enhance immune responses by stimulating macrophages, dendritic cells, and B lymphocytes [23,28,62], which may explain the maintained ND titers in CMD birds.

### 3.4. Liver enzymes

Neither mycotoxin contamination nor CMD supplementation significantly affected liver enzyme activities (ALT, AST, and GGT) on days 15 and 37 (Table 7). This indicates that the mycotoxin concentrations used in the present study did not induce severe hepatocellular injury sufficient to alter circulating liver enzyme activities. Typically, elevated ALT and AST reflect hepatocellular damage, whereas GGT is an indicator of hepatobiliary stress. Although aflatoxins are well known to induce hepatotoxicity through oxidative stress, lipid peroxidation, and apoptosis of hepatocytes [13,16], the extent of liver injury depends on the dose and duration of exposure. In the present study, the absence of significant changes in liver enzymes despite histopathological alterations suggests that the lesions were mild or localized and therefore insufficient to cause substantial enzyme leakage into the bloodstream. Histopathological examination is generally more sensitive than serum biochemical markers for detecting early or subclinical hepatic injury and can reveal structural alterations before functional impairment becomes evident. Thus, the observed histological changes likely represent early-stage hepatic responses to chronic low-level mycotoxin exposure rather than overt liver dysfunction. Moreover, CMD supplementation did not adversely affect liver enzyme activities, reinforcing its safety. The sodium bentonite, sepiolite, yeast cell wall, and β-glucan components of CMD likely reduced gastrointestinal mycotoxin absorption, thereby limiting hepatic exposure and minimizing progression from localized tissue alterations to clinically detectable liver injury.

**Table 7:** Liver enzyme activities in broilers fed mycotoxin-contaminated diet and supplemented with a complex mycotoxin detoxifier.

Items	Dietary treatment^1^			SEM	P value
	PC	NC	CMD		
15 Day					
ALT (U/L)	3.71	3.94	3.56	0.08	0.164
AST (U/L)	182.81	190.31	180.14	2.58	0.307
GGT (U/L)	11.94	12.13	10.44	0.49	0.348
37 Day					
ALT (U/L)	3.75	3.81	3.19	0.13	0.175
AST (U/L)	298.56	298.81	284.69	11.64	0.620
GGT (U/L)	21.07	21.94	18.94	0.70	0.310

^1^PC: basal diet without mycotoxin contamination; NC: basal diet contaminated with 80 ppb Aflatoxin + 900 ppb fumonisins + 30 ppb deoxynivalenol + 80 ppb Zearalenone; CMD: NC + complex mycotoxin detoxifier. AST, Aspartate aminotransferase, ALT, Alanine aminotransferase, GGT, gamma- glutamyl transferase.

### 3.5. Liver health

**Fig 2:**
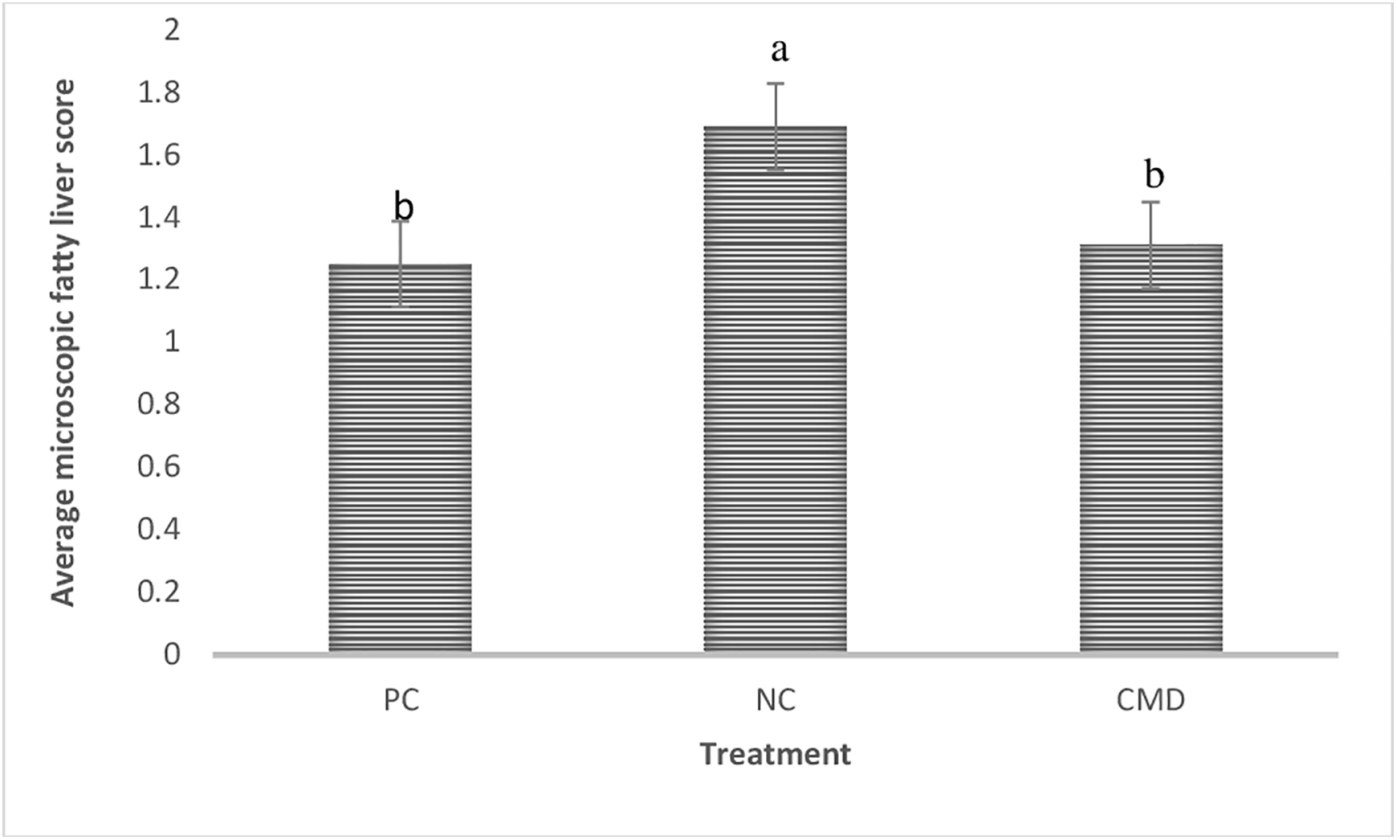
Average fatty liver scores in broilers fed mycotoxin-contaminated diet and supplemented with a complex mycotoxin detoxifier. ^1^PC: basal diet without mycotoxin contamination; NC: basal diet contaminated with 80 ppb Aflatoxin + 900 ppb fumonisins + 30 ppb deoxynivalenol + 80 ppb Zearalenone; CMD: NC + complex mycotoxin detoxifier.

**Fig 3:**
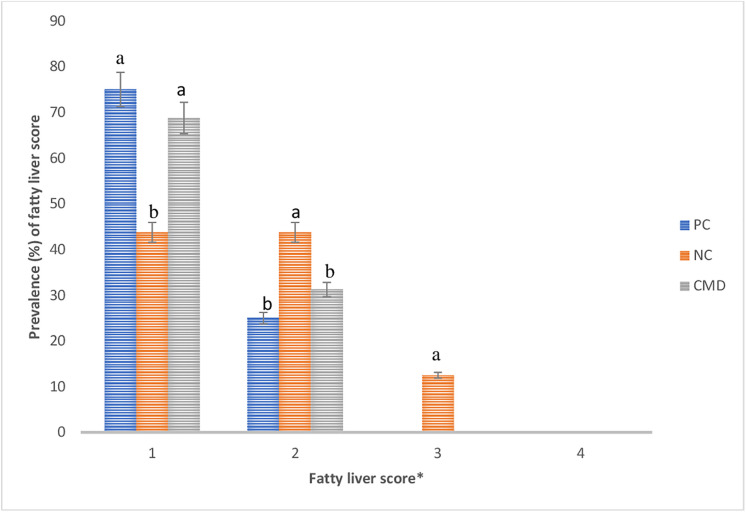
Prevalence of fatty liver score in broilers fed mycotoxin-contaminated diet and supplemented with a complex mycotoxin detoxifier. ^1^PC: basal diet without mycotoxin contamination; NC: basal diet contaminated with 80 ppb Aflatoxin + 900 ppb fumonisins + 30 ppb deoxynivalenol + 80 ppb Zearalenone; CMD: NC + complex mycotoxin detoxifier. Score 1: normal liver with dark red. Score 2: mild case of fatty liver hemorrhagic syndrome (FLHS) with mild yellow liver and hemorrhages. Score 3: moderate case of FLHS, light yellowish red liver and hemorrhages. Score 4: severe case of FLHS, large and massive hemorrhages with putty-colored livers.

The average fatty liver score was significantly higher in NC birds (1.69) than in PC (1.25) and CMD (1.31) birds (Fig 2), indicating that mycotoxin exposure increased the severity of fatty liver hemorrhagic syndrome. Although severe fatty liver cases (Scores 3 and 4) were rare across treatments (Fig 3), Score 3 occurred more frequently in NC birds (12.5%) than in PC (0%) and CMD (0%) birds, confirming that mycotoxin contamination exacerbated hepatic injury. The lower fatty liver score in CMD birds demonstrates that the detoxifier mitigated, but did not completely prevent, hepatic steatosis.

**Fig 4:**
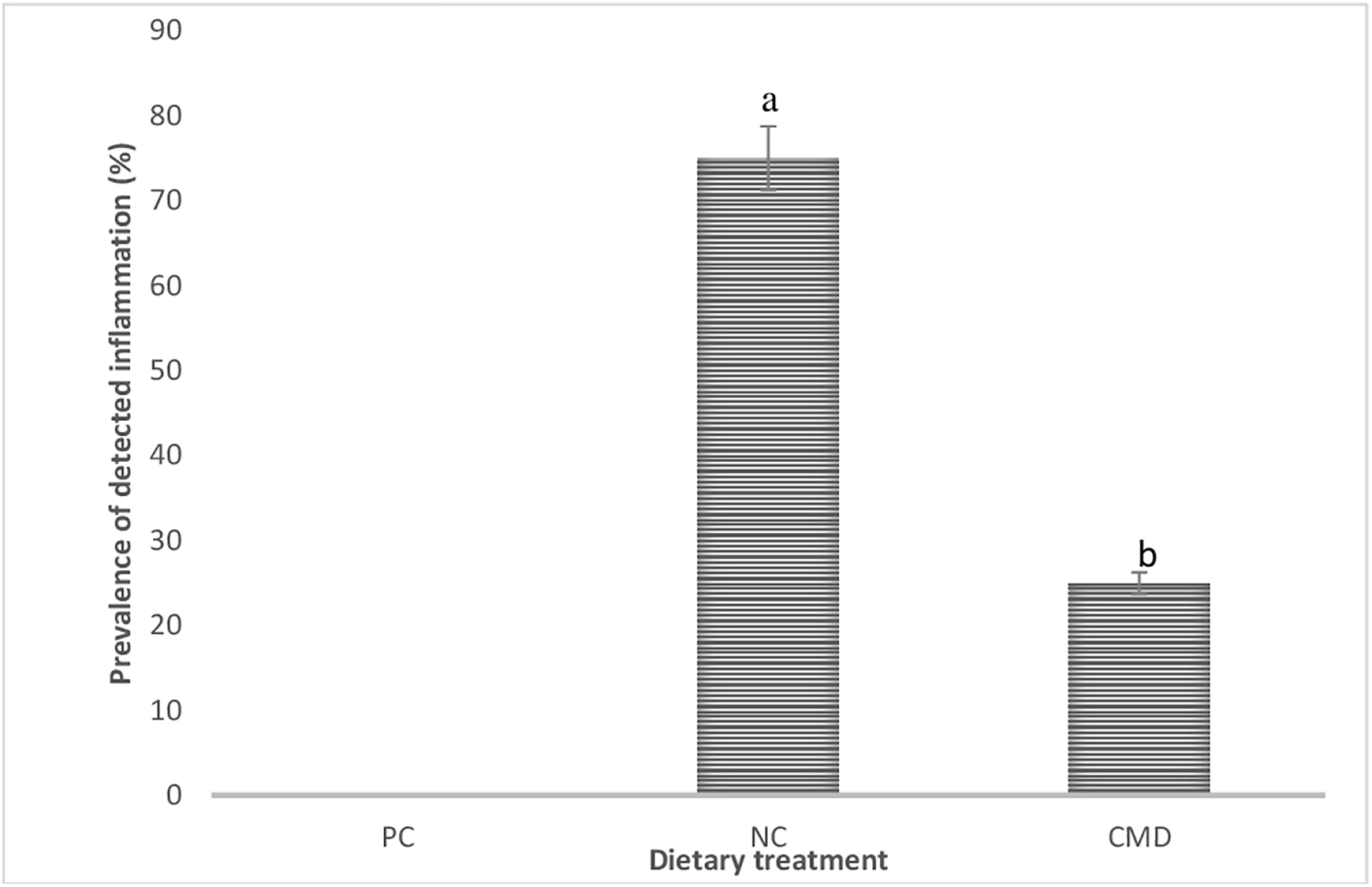
Prevalence (%) of detected liver inflammation in broilers fed mycotoxin-contaminated diet and supplemented with a complex mycotoxin detoxifier. ^1^PC: basal diet without mycotoxin contamination; NC: basal diet contaminated with 80 ppb Aflatoxin + 900 ppb fumonisins + 30 ppb deoxynivalenol + 80 ppb Zearalenone; CMD: NC + complex mycotoxin detoxifier.

Mycotoxin contamination also significantly increased liver inflammation in NC birds (75.0%) compared with PC (0.0%) and CMD (25.0%) birds (Fig 4). These findings confirm that chronic low-level mycotoxin exposure induces hepatic inflammation and steatosis, consistent with previous reports [16,62]. The increased fatty liver and inflammation scores likely reflect disruption of hepatic lipid metabolism and oxidative homeostasis, resulting in impaired lipid transport and excessive lipid accumulation within hepatocytes [27,63,64] as previously reported in broilers exposed to ochratoxin A, deoxynivalenol, and aflatoxin B1 [27] and mixed mycotoxins [63]. By reducing gastrointestinal absorption of mycotoxins through its clay mineral components and supporting intestinal health and immune function via yeast-derived β-glucans and cell wall fractions, CMD likely attenuated these pathological processes and improved hepatic resilience.

Nevertheless, CMD did not fully restore liver health to the level observed in the positive control. This partial protection may be explained by incomplete adsorption or detoxification of multiple co-occurring mycotoxins, allowing a fraction of toxins to remain bioavailable. In addition, chronic exposure to several mycotoxins may produce additive or synergistic hepatotoxic effects that cannot be entirely counteracted by a single detoxification strategy. Consequently, while CMD substantially reduced the severity of hepatic lesions, residual histopathological alterations persisted in some birds, indicating that detoxification alleviated rather than completely reversed mycotoxin-induced liver injury. Consistent with these results, yeast polysaccharides [17] and complex mycotoxin detoxifiers [12,13] have been shown to alleviate hepatic damage and inflammation in broilers fed mycotoxin-contaminated diets.

## 4. Conclusions

This study demonstrated that chronic low-level mycotoxin contamination impaired growth efficiency, humoral immune response, and liver health in broilers, despite having minimal effects on systemic inflammatory and oxidative stress biomarkers. Supplementation with a complex mycotoxin detoxifier partially mitigated these adverse effects by preserving Newcastle disease antibody titers and reducing hepatic steatosis and inflammation, although complete restoration of liver health was not achieved. These findings highlight the potential of complex mycotoxin detoxifiers to improve broiler resilience under subclinical mycotoxin exposure while emphasizing that residual hepatic alterations may persist. Future studies should investigate strategies to enhance detoxifier efficacy against multi-mycotoxin challenges under diverse commercial production conditions.
